# Supplementary material for: Bioinformatics analysis of the structural and evolutionary characteristics for toll-like receptor 15
Source: PeerJ. 2016 May 25;4:e2079. doi: 10.7717/peerj.2079 (PMC4888287; doi:10.7717/peerj.2079)
Supplement: Table S1 [file peerj-04-2079-s001.docx]

**Supplemental Table S1.** **Information of the completed TLR15 gene sequences from avian and reptilian species.**

| **No.** | **Taxon** | **Scientific Name** | **Accession No.** | **Length (bp)** |
| --- | --- | --- | --- | --- |
| 1 | Aves | *Anas platyrhynchos* | XM_005018870 | 2595 |
| 2 | Aves | *Anser anser* | JQ014619 | 2598 |
| 3 | Aves | *Apaloderma vittatum* | XM_009870709 | 2616 |
| 4 | Aves | *Aptenodytes forsteri* | XM_009288440 | 2625 |
| 5 | Aves | *Balearica regulorum gibbericeps* | XM_010298512 | 2625 |
| 6 | Aves | *Buceros rhinoceros silvestris* | XM_010142512 | 2613 |
| 7 | Aves | *Calypte anna* | XM_008493258 | 2628 |
| 8 | Aves | *Caprimulgus carolinensis* | XM_010174731 | 2622 |
| 9 | Aves | *Cariama cristata* | XM_009706095 | 2625 |
| 10 | Aves | *Chaetura pelagica* | XM_010004652 | 2625 |
| 11 | Aves | *Charadrius vociferus* | XM_009886001 | 2625 |
| 12 | Aves | *Chlamydotis macqueenii* | XM_010125500 | 2625 |
| 13 | Aves | *Colius striatus* | XM_010206127 | 2577 |
| 14 | Aves | *Columba livia* | XM_005513180 | 2517 |
| 15 | Aves | *Corvus brachyrhynchos* | XM_008642044 | 2625 |
| 16 | Aves | *Corvus cornix cornix* | XM_010402971 | 2625 |
| 17 | Aves | *Coturnix coturnix* | HM773176 | 2607 |
| 18 | Aves | *Cuculus canorus* | XM_009560965 | 2625 |
| 19 | Aves | *Egretta garzetta* | XM_009640695 | 2625 |
| 20 | Aves | *Eurypyga helias* | XM_010151478 | 2616 |
| 21 | Aves | *Falco cherrug* | XM_005445628 | 2622 |
| 22 | Aves | *Falco peregrinus* | XM_005235036 | 2619 |
| 23 | Aves | *Ficedula albicollis* | ENSFALG00000015347 | 2628 |
| 24 | Aves | *Fulmarus glacialis* | XM_009586905 | 2625 |
| 25 | Aves | *Gallus gallus* | NM_001037835 | 2607 |
| 26 | Aves | *Gallus lafayetii* | FJ915220 | 2607 |
| 27 | Aves | *Gallus sonneratii* | FJ915243 | 2607 |
| 28 | Aves | *Gavia stellata* | XM_009821678 | 2625 |
| 29 | Aves | *Geospiza fortis* | XM_005422603 | 2625 |
| 30 | Aves | *Haliaeetus albicilla* | XM_009927303 | 2622 |
| 31 | Aves | *Haliaeetus leucocephalus* | XM_010574879 | 2625 |
| 32 | Aves | *Leptosomus discolor* | XM_009953779 | 2625 |
| 33 | Aves | *Manacus vitellinus* | XM_008924845 | 2634 |
| 34 | Aves | *Meleagris gallopavo* | ENSMGAG00000015891 | 2604 |
| 35 | Aves | *Melopsittacus undulatus* | XM_005144697 | 2622 |
| 36 | Aves | *Merops nubicus* | XM_008943754 | 2610 |
| 37 | Aves | *Mesitornis unicolor* | XM_010182944 | 2625 |
| 38 | Aves | *Nestor notabilis* | XM_010013070 | 2622 |
| 39 | Aves | *Nipponia nippon* | XM_009476126 | 2625 |
| 40 | Aves | *Opisthocomus hoazin* | XM_009931299 | 2625 |
| 41 | Aves | *Pelecanus crispus* | XM_009492835 | 2628 |
| 42 | Aves | *Phaethon lepturus* | XM_010282406 | 2613 |
| 43 | Aves | *Phalacrocorax carbo* | XM_009507639 | 2625 |
| 44 | Aves | *Picoides pubescens* | XM_009902092 | 2556 |
| 45 | Aves | *Pseudopodoces humilis* | XM_005525475 | 2625 |
| 46 | Aves | *Pterocles gutturalis* | XM_010085978 | 2673 |
| 47 | Aves | *Pygoscelis adeliae* | XM_009319611 | 2625 |
| 48 | Aves | *Serinus canaria* | XM_009093733 | 2586 |
| 49 | Aves | *Struthio camelus australis* | XM_009669051 | 2625 |
| 50 | Aves | *Taeniopygia guttata* | XM_002197069 | 2625 |
| 51 | Aves | *Tauraco erythrolophus* | XM_009979140 | 2622 |
| 52 | Aves | *Tinamus guttatus* | XM_010218127 | 2637 |
| 53 | Aves | *Tyto alba* | XM_009975442 | 2616 |
| 54 | Aves | *Zonotrichia albicollis* | XM_005486787 | 2625 |
| 55 | Reptiles | *Alligator mississippiensis* | XM_006274445 | 2628 |
| 56 | Reptiles | *Alligator sinensis* | XM_006021588 | 2628 |
| 57 | Reptiles | *Python bivittatus* | XM_007444506 | 2169 |
