# Supplementary material for: Bioinformatics analysis of the structural and evolutionary characteristics for toll-like receptor 15
Source: PeerJ. 2016 May 25;4:e2079. doi: 10.7717/peerj.2079 (PMC4888287; doi:10.7717/peerj.2079)
Supplement: Table S2 [file peerj-04-2079-s002.docx]

**Supplemental Table S2. Amino acid compositions in the asparagine ladder positions of avian TLR ectodomains.**

| **Scientific Name** | **Accession No.** | **LRR module** | | | | | | | | | | | | | | | | | | | | | | | | |
| --- | --- | --- | --- | --- | --- | --- | --- | --- | --- | --- | --- | --- | --- | --- | --- | --- | --- | --- | --- | --- | --- | --- | --- | --- | --- | --- |
|  |  | **1** | **2** | **3** | **4** | **5** | **6** | **7** | **8** | **9** | **10** | **11** | **12** | **13** | **14** | **15** | **16** | **17** | **18** | **19** | **20** | **21** | **22** | **23** | **24** | **25** |
| **TLR1LA** | | | | | | | | | | | | | | | | | | | | | | | | | | |
| *Anas_platyrhynchos* | ENSAPLG00000002049 | N | N | N | N | T | E | S | T | V | L | V | A | N | N | N | N | N | N | N |  |  |  |  |  |  |
| *Falco_cherrug* | XM_005435252 | N | N | N | N | T | E | A | T | V | L | V | A | N | N | N | N | N | N | N |  |  |  |  |  |  |
| *Falco_peregrinus* | XM_005243208 | N | N | N | N | T | E | A | T | V | L | V | A | N | N | N | N | N | N | N |  |  |  |  |  |  |
| *Ficedula_albicollis* | ENSFALG00000014902 | N | N | N | N | T | E | A | T | V | L | V | A | N | N | N | N | N | N | N |  |  |  |  |  |  |
| *Gallus_gallus* | ENSGALG00000017485 | N | N | N | N | M | E | A | T | V | L | V | A | N | N | N | N | N | N | N |  |  |  |  |  |  |
| *Geospiza_fortis* | XM_005431639 | N | N | N | N | T | G | A | T | V | L | V | A | N | N | N | N | N | N | N |  |  |  |  |  |  |
| *Meleagris_gallopavo* | FJ477857 | N | N | N | N | T | E | A | T | G | L | V | A | N | N | N | N | N | N | N |  |  |  |  |  |  |
| *Pseudopodoces_humilis* | XM_005518036 | N | N | N | N | T | R | A | T | V | L | V | A | N | N | N | N | N | N | N |  |  |  |  |  |  |
| *Taeniopygia_guttata* | XM_004176605 | N | N | N | N | T | G | A | T | V | L | V | A | N | N | N | N | N | N | N |  |  |  |  |  |  |
| **TLR1LB** | | | | | | | | | | | | | | | | | | | | | | | | | | |
| *Anas_platyrhynchos* | JN572686 | - | - | - | - | - | - | - | I | V | F | I | S | N | N | N | N | N | N | N |  |  |  |  |  |  |
| *Falco_cherrug* | XM_005435373 | - | - | - | - | - | - | - | I | V | I | V | S | N | N | N | N | N | N | N |  |  |  |  |  |  |
| *Falco_peregrinus* | XM_005243247 | - | - | - | - | - | - | - | I | V | I | V | S | N | N | N | N | N | N | N |  |  |  |  |  |  |
| *Ficedula_albicollis* | ENSFALG00000014882 | - | - | - | - | - | - | - | I | V | V | I | S | N | N | N | N | N | N | N |  |  |  |  |  |  |
| *Gallus_gallus* | ENSGALG00000027093 | - | - | - | - | - | - | - | I | M | L | V | S | N | N | N | N | N | N | N |  |  |  |  |  |  |
| *Geospiza_fortis* | XM_005415453 | - | - | - | - | - | - | - | I | V | V | I | S | N | N | N | N | N | N | N |  |  |  |  |  |  |
| *Meleagris_gallopavo* | FJ477858 | - | - | - | - | - | - | - | I | M | L | V | S | N | N | N | N | N | N | N |  |  |  |  |  |  |
| *Pseudopodoces_humilis* | XM_005518035 | - | - | - | - | - | - | - | I | V | V | I | S | N | N | N | N | N | N | N |  |  |  |  |  |  |
| *Taeniopygia_guttata* | ENSTGUG00000009041 | - | - | - | - | - | - | - | I | V | V | I | S | N | N | N | N | N | N | N |  |  |  |  |  |  |
| **TLR2A** | | | | | | | | | | | | | | | | | | | | | | | | | | |
| *Anas_platyrhynchos* | ENSAPLG00000011397 | N | N | N | N | S | S | I | D | K | S | M | S | N | N | N | T | N | N | N |  |  |  |  |  |  |
| *Falco_cherrug* | XM_005436665 | N | N | N | N | S | G | I | E | Q | C | I | S | N | N | N | T | N | N | N |  |  |  |  |  |  |
| *Falco_peregrinus* | XM_005232499 | N | N | N | N | S | G | I | E | Q | C | I | S | N | N | N | T | N | N | N |  |  |  |  |  |  |
| *Ficedula_albicollis* | ENSFALG00000013087 | N | N | N | N | S | S | I | P | Q | C | I | S | N | N | N | T | N | N | N |  |  |  |  |  |  |
| *Gallus_gallus* | XM_003641158 | N | N | N | N | S | D | I | D | K | C | M | S | N | N | N | T | N | N | N |  |  |  |  |  |  |
| *Geospiza_fortis* | XM_005415589 | N | N | N | N | S | S | L | P | Q | C | A | S | N | N | N | T | N | N | N |  |  |  |  |  |  |
| *Meleagris_gallopavo* | ENSMGAG00000001271 | N | N | N | N | S | G | V | D | Q | C | M | S | N | N | N | T | N | N | N |  |  |  |  |  |  |
| *Pseudopodoces_humilis* | XM_005517566 | N | N | N | N | S | S | L | P | Q | C | I | S | N | N | N | T | N | N | N |  |  |  |  |  |  |
| *Taeniopygia_guttata* | ENSTGUG00000005179 | N | N | N | N | S | S | L | P | E | C | I | S | N | N | N | T | N | N | N |  |  |  |  |  |  |
| **TLR2B** | | | | | | | | | | | | | | | | | | | | | | | | | | |
| *Anas_platyrhynchos* | ENSAPLG00000011399 | N | N | N | N | S | S | I | N | A | C | L | T | N | N | N | T | N | N | N |  |  |  |  |  |  |
| *Falco_cherrug* | XM_005436664 | N | N | N | N | S | G | T | S | A | C | L | T | N | N | N | T | N | N | N |  |  |  |  |  |  |
| *Falco_peregrinus* | XM_005232498 | N | N | N | N | S | G | T | S | A | C | L | T | N | N | N | T | N | N | N |  |  |  |  |  |  |
| *Ficedula_albicollis* | ENSFALG00000014085 | N | N | N | N | S | S | I | S | A | C | L | T | N | N | N | T | N | N | N |  |  |  |  |  |  |
| *Gallus_gallus* | AB046533 | N | N | N | N | S | D | I | S | A | C | L | T | N | N | N | T | N | N | N |  |  |  |  |  |  |
| *Geospiza_fortis* | XM_005415709 | N | N | N | N | S | S | I | S | A | C | L | T | N | N | N | T | N | N | N |  |  |  |  |  |  |
| *Meleagris_gallopavo* | FJ477861 | N | N | N | N | S | G | V | S | A | C | L | T | N | N | N | T | N | N | N |  |  |  |  |  |  |
| *Pseudopodoces_humilis* | XM_005517567 | N | N | N | N | S | S | I | G | A | S | L | T | N | N | N | T | N | N | N |  |  |  |  |  |  |
| *Taeniopygia_guttata* | XM_002196366 | N | N | N | N | S | S | L | S | A | C | L | T | N | N | N | T | N | N | N |  |  |  |  |  |  |
| **TLR3** | | | | | | | | | | | | | | | | | | | | | | | | | | |
| *Anas_platyrhynchos* | ENSAPLG00000008976 | N | N | N | N | N | N | N | N | S | N | N | S | N | N | T | N | N | V | N | N | N | N | N |  |  |
| *Falco_cherrug* | XM_005433774 | N | N | N | N | N | N | N | N | S | N | N | S | N | N | T | N | N | V | N | N | N | N | N |  |  |
| *Falco_peregrinus* | XM_005243141 | N | N | N | N | N | N | N | N | S | N | N | S | N | N | T | N | N | V | N | N | N | N | N |  |  |
| *Ficedula_albicollis* | ENSFALG00000011936 | N | N | N | N | N | N | N | N | S | N | N | S | N | N | T | N | N | V | N | N | N | N | N |  |  |
| *Gallus_gallus* | ENSGALG00000013468 | N | N | N | N | N | N | N | N | S | N | N | S | N | N | T | N | N | V | N | N | N | N | N |  |  |
| *Geospiza_fortis* | XM_005415516 | N | N | N | N | N | N | N | N | S | N | N | S | N | N | T | N | N | V | N | N | N | N | N |  |  |
| *Meleagris_gallopavo* | ENSMGAG00000011425 | N | N | N | N | N | N | N | N | S | N | N | A | N | N | T | N | N | V | N | N | N | N | N |  |  |
| *Pseudopodoces_humilis* | XM_005518005 | N | N | N | N | N | N | N | N | S | N | N | S | N | N | T | N | N | V | N | N | N | N | N |  |  |
| *Taeniopygia_guttata* | XM_002190852 | N | N | N | N | N | N | N | N | S | N | N | S | N | N | T | N | N | V | N | N | N | N | N |  |  |
| **TLR4** | | | | | | | | | | | | | | | | | | | | | | | | | | |
| *Anas_platyrhynchos* | ENSAPLG00000012625 | N | C | N | T | N | N | N | S | F | F | V | C | S | N | N | T | T | N | C | N | N |  |  |  |  |
| *Falco_cherrug* | XM_005441990 | N | C | N | T | N | N | N | S | F | V | L | C | N | N | N | T | T | N | C | N | N |  |  |  |  |
| *Falco_peregrinus* | XM_005231393 | N | C | N | T | N | N | N | S | F | V | L | C | N | N | N | T | T | N | C | N | N |  |  |  |  |
| *Ficedula_albicollis* | ENSFALG00000007733 | S | C | N | T | N | N | N | A | F | S | L | C | N | N | N | T | T | N | C | N | N |  |  |  |  |
| *Gallus_gallus* | ENSGALG00000007001 | N | C | N | T | N | N | N | S | F | F | L | C | S | N | N | T | T | N | C | N | N |  |  |  |  |
| *Geospiza_fortis* | XM_005423810 | S | C | N | T | N | N | N | A | F | L | L | C | N | N | N | T | T | N | C | N | N |  |  |  |  |
| *Meleagris_gallopavo* | ENSMGAG00000005422 | N | C | N | T | N | N | N | S | F | F | L | C | S | N | N | T | T | N | C | N | N |  |  |  |  |
| *Pseudopodoces_humilis* | XM_005527197 | S | C | N | T | N | N | N | A | F | F | L | C | N | N | N | T | T | N | C | N | N |  |  |  |  |
| *Taeniopygia_guttata* | NM_001142454 | S | C | N | T | N | N | N | A | F | L | L | C | N | N | N | T | T | N | C | N | N |  |  |  |  |
| **TLR5** | | | | | | | | | | | | | | | | | | | | | | | | | | |
| *Anas_platyrhynchos* | ENSAPLG00000001279 | N | Q | N | N | N | N | T | S | H | G | N | N | N | N | N | N | N | N | N | N | N |  |  |  |  |
| *Falco_cherrug* | XM_005441254 | N | Q | N | N | N | N | N | N | H | G | N | N | N | N | N | N | N | N | N | N | N |  |  |  |  |
| *Falco_peregrinus* | XM_005241848 | N | Q | N | N | N | N | N | N | H | G | N | N | N | N | N | N | N | N | N | N | N |  |  |  |  |
| *Ficedula_albicollis* | ENSFALG00000014401 | N | Q | N | N | N | N | N | S | H | G | N | N | N | N | N | N | N | N | N | N | N |  |  |  |  |
| *Gallus_gallus* | ENSGALG00000009392 | N | Q | N | N | N | N | N | N | H | G | N | N | N | N | N | N | N | N | N | N | N |  |  |  |  |
| *Meleagris_gallopavo* | ENSMGAG00000015929 | N | Q | N | N | N | N | N | N | H | G | N | N | N | N | N | N | N | N | N | N | N |  |  |  |  |
| *Pseudopodoces_humilis* | XM_005523308 | N | Q | N | N | N | N | N | N | H | G | N | N | N | N | N | N | N | N | N | N | N |  |  |  |  |
| *Taeniopygia_guttata* | ENSTGUG00000002653 | N | Q | N | N | N | N | N | S | T | G | N | N | N | N | N | N | N | N | N | N | N |  |  |  |  |
| **TLR7** | | | | | | | | | | | | | | | | | | | | | | | | | | |
| *Anas_platyrhynchos* | ENSAPLG00000004139 | N | N | N | N | N | N | N | N | N | N | N | Y | N | N | N | N | N | N | N | N | N | N | N | N | N |
| *Falco_cherrug* | XM_005434509 | N | N | N | N | N | N | N | N | N | N | N | Y | N | N | N | N | N | N | N | N | N | N | N | N | N |
| *Falco_peregrinus* | XM_005229443 | N | N | N | N | N | N | N | N | N | N | N | Y | N | N | N | N | N | N | N | N | N | N | N | N | N |
| *Ficedula_albicollis* | ENSFALG00000004472 | N | N | N | N | N | N | N | N | N | N | N | Y | N | N | N | N | N | N | N | N | N | N | N | N | N |
| *Gallus_gallus* | ENSGALG00000016590 | N | N | N | N | N | N | N | N | N | N | N | Y | N | N | N | N | N | N | N | N | N | N | N | N | N |
| *Geospiza_fortis* | XM_005429715 | N | N | N | N | N | N | N | N | N | N | N | Y | N | N | N | N | N | N | N | N | N | N | N | N | N |
| *Meleagris_gallopavo* | ENSMGAG00000014706 | N | N | N | N | N | N | N | N | N | N | N | Y | N | N | N | N | N | N | N | N | N | N | N | N | N |
| *Pseudopodoces_humilis* | XM_005516845 | N | N | N | N | N | N | N | N | N | N | N | Y | N | N | N | N | N | N | N | N | N | N | N | N | N |
| *Taeniopygia_guttata* | XM_002194896 | N | N | N | N | N | N | N | N | N | N | N | Y | N | N | N | N | N | N | N | N | N | N | N | N | N |
| **TLR15** | | | | | | | | | | | | | | | | | | | | | | | | | | |
| *Anas_platyrhynchos* | XM_005018870 | N | N | N | S | N | N | N | T | N | N | C | N | S | N | N | N | N | N | N |  |  |  |  |  |  |
| *Falco_cherrug* | XM_005445628 | N | N | N | S | S | N | N | T | N | N | C | N | S | N | N | N | N | N | N |  |  |  |  |  |  |
| *Falco_peregrinus* | XM_005235036 | N | N | N | S | S | N | N | T | N | N | C | N | S | N | N | N | N | N | N |  |  |  |  |  |  |
| *Ficedula_albicollis* | ENSFALG00000015347 | N | N | N | S | N | N | N | T | N | N | C | N | N | N | N | N | N | N | N |  |  |  |  |  |  |
| *Gallus_gallus* | NM_001037835 | N | N | N | T | S | N | N | T | N | N | C | N | S | N | N | N | N | N | N |  |  |  |  |  |  |
| *Geospiza_fortis* | XM_005422603 | N | N | N | S | N | N | N | T | N | K | C | N | N | N | N | N | N | N | N |  |  |  |  |  |  |
| *Meleagris_gallopavo* | ENSMGAG00000015891 | N | N | N | T | S | N | N | T | N | N | C | N | S | N | N | N | N | N | N |  |  |  |  |  |  |
| *Pseudopodoces_humilis* | XM_005525475 | N | N | N | S | N | N | N | T | N | N | C | N | N | N | N | N | N | N | N |  |  |  |  |  |  |
| *Taeniopygia_guttata* | XM_002197069 | N | N | N | S | N | N | N | T | N | N | C | N | N | N | N | N | N | N | N |  |  |  |  |  |  |
| **TLR21** | | | | | | | | | | | | | | | | | | | | | | | | | | |
| *Falco_peregrinus* | XM_005238572 | N | N | N | N | G | N | N | N | N | G | N | N | - | N | N | N | N | N | N | N | Q | N | S | N | N |
| *Ficedula_albicollis* | XM_005062396 | N | N | N | N | G | N | N | N | N | G | L | N | N | N | N | N | N | N | N | N | Q | N | S | S | N |
| *Gallus_gallus* | ENSGALG00000000774 | N | N | N | N | G | N | N | N | N | Q | N | N | N | N | N | N | N | N | N | N | Q | N | S | N | N |
| *Geospiza_fortis* | XM_005431221 | N | N | N | N | G | N | N | N | N | S | M | N | - | - | N | N | N | N | N | N | Q | N | S | S | N |
| *Meleagris_gallopavo* | ENSMGAG00000015581 | N | N | N | N | G | N | N | N | N | H | N | N | N | N | N | N | N | N | N | N | Q | N | S | N | N |
| *Pseudopodoces_humilis* | XM_005533956 | N | N | N | N | G | N | N | N | N | G | M | N | N | N | N | N | N | N | N | N | Q | N | S | S | N |
